# The effect of aqueous extract of Iranian oak (*Quercus brantii*) on lipid profile and liver enzymes in beta-thalassemia patients: a randomized controlled trial, double-blind, placebo-controlled

**DOI:** 10.3389/fnut.2025.1537420

**Published:** 2025-03-26

**Authors:** Mahdi Babamir Satehi, Mahdi Karimi, Ataollah Eskandari, Hamid Mahmoodi

**Affiliations:** ^1^Clinical Biochemistry Research Center, Shahrekord University of Medical Sciences, Shahrekord, Iran; ^2^Nutrition and Metabolic Diseases Research Center, Clinical Sciences Research Institute, Ahvaz Jundishapur University of Medical Sciences, Ahvaz, Iran; ^3^Department of Nutrition, School of Allied Medical Sciences, Ahvaz Jundishapur University of Medical Sciences, Ahvaz, Iran; ^4^Student Research Committee, Ahvaz Jundishapur University of Medical Sciences, Ahvaz, Iran; ^5^Student Research Committee, Tabriz University of Medical Sciences, Tabriz, Iran; ^6^Student Research Committee, Shiraz University of Medical Sciences, Shiraz, Iran

**Keywords:** thalassemia, lipid profile, liver enzymes, oak extract, antioxidant therapy

## Abstract

**Background and aim:**

Beta-thalassemia major patients often require regular blood transfusions, leading to iron overload, oxidative stress, and disturbances in lipid metabolism. The common use of vitamins and iron chelators helps mitigate some of these effects, but lipid profile abnormalities persist. The oak fruit (*Quercus brantii*) is rich in antioxidant compounds, such as flavonoids and polyphenols, which may help address these issues. This study aimed to investigate the effects of the aqueous extract of Iranian oak on the lipid profile and liver enzymes in beta-thalassemia major patients.

**Materials and methods:**

This randomized, double-blind, placebo-controlled clinical trial included 60 beta-thalassemia major patients (29 males, 31 females; age 10–60 years) who regularly received blood transfusions and deferoxamine. Participants were divided into two groups: the intervention group received Iranian oak extract capsules (300 mg/day), and the control group received placebo capsules for 3 months. Lipid profiles (cholesterol, triglycerides, HDL, LDL) and liver enzymes (ALT, AST) were measured before and after the intervention.

**Results:**

The control group exhibited a significant increase in triglyceride levels (from 167 to 184 mg/dL, *p* < 0.03), while no significant changes were observed in the intervention group. In contrast, total cholesterol significantly decreased in the oak extract group (from 125 to 112 mg/dL, *p* < 0.003). HDL levels decreased in both groups (*p* = 0.008 for the intervention group; *p* = 0.016 for the control group). No significant differences were found in LDL, ALT, or AST levels between the two groups.

**Conclusion:**

The aqueous extract of Iranian oak demonstrated potential lipid-modulating effects by preventing triglyceride increases and reducing cholesterol levels in beta-thalassemia major patients. These findings suggest that the antioxidant properties of the oak extract may help manage lipid abnormalities associated with iron overload, improving cardiovascular risk profiles in these patients. Further studies with larger sample sizes and extended follow-up are recommended to confirm these benefits.

**Clinical trial tegistration:**

http://www.irct.ir, identifier IRCT2015101411819N4.

## 1 Introduction

Thalassemia is the most prevalent hereditary hemoglobinopathy globally (1, 2). It arises due to defects in the production of the alpha or beta chains of hemoglobin. Beta-thalassemia specifically involves abnormalities in the beta chain, which results in various clinical manifestations, ranging from minor to major thalassemia depending on the severity of the anemia and the need for blood transfusions (3). A “thalassemia belt” exists globally, extending from Southern Europe and Northern Africa through the Middle East to the Far East, with Iran also falling within this region. Approximately 3 million individuals in Iran are estimated to be carriers of thalassemia, and around 20,000 individuals are affected by thalassemia major, with a higher prevalence in the northern provinces and the southern coastal regions (4, 5).

In beta-thalassemia major, iron overload occurs primarily due to two mechanisms: increased intestinal iron absorption (resulting from ineffective erythropoiesis to compensate for impaired oxygen delivery) and frequent blood transfusions (prolonging lifespan and durability) (6, 7). Evidence indicates that oxidative stress caused by iron accumulation is a key factor in the pathogenicity of iron overload in thalassemia (8, 9). Complications associated with iron overload include endocrine disorders, cardiac and hepatic failure, infections, and lipid metabolism abnormalities (10, 11). Hypertriglyceridemia and hypocholesterolemia are the most frequently reported lipid disorders in thalassemia. Separate studies by Amendola, Mona, and Kamal have documented hypocholesterolemia and hypertriglyceridemia in patients with thalassemia, attributing cholesterol reduction to increased consumption by the bone marrow and triglyceride elevation to heightened oxidative stress (12–14).

While previous studies have documented lipid profile abnormalities in patients with beta-thalassemia major, the precise mechanisms underlying these abnormalities remain unclear. Several reports suggest mechanisms such as increased free radicals, enhanced oxidation and fatty acid production, elevated erythropoiesis, impaired liver function, activation of the macrophage system by cytokines, and hormonal disturbances due to iron overload as contributors to lipid abnormalities in beta-thalassemia major patients (3, 12, 13). The absorption and production of triglycerides (TG) and cholesterol, along with elevated oxidative substances, are directly associated with lipid abnormalities in thalassemia patients (15). Pancreatic lipase plays a crucial role in triglyceride digestion and absorption in the small intestine, while HMG-CoA reductase is involved in endogenous cholesterol production (16, 17).

A wide range of plant compounds, including tannins and phenolic compounds, have been shown to inhibit enzymes like lipase and HMG-CoA reductase (18, 19). Oak (Quercus spp.) has long been used as an antifungal, antimicrobial, and in the treatment of hemorrhoids and burns. In Europe, especially in Mediterranean countries like Italy and Spain, oak fruit consumption constitutes about 25% of the food basket and is commonly available in supermarkets (20). Oak fruit is rich in active flavonoids and phenolic compounds such as tannins and gallic acid, which possess antioxidant properties and high protein content (21–24). Numerous laboratory studies have demonstrated that oak significantly enhances antioxidant capacity due to its phenolic content (25, 26). Moreover, oak has been shown to inhibit cholesterol and triglyceride production and absorption (27, 28).

We hypothesize that the aqueous extract of Iranian oak (*Quercus brantii*), due to its antioxidant properties, may improve lipid profile and regulate liver enzyme levels in beta-thalassemia patients.

Although substantial research has been conducted on thalassemia and the endocrine system, the number of studies in Iran focusing on lipid profiles and supplements consumed by thalassemia patients remains limited. This study aims to evaluate the lipid profile and the effect of an aqueous extract of oak in patients with beta-thalassemia, considering the therapeutic properties of oak.

## 2 Materials and methods

### 2.1 Patients

The present study enrolled participants from the Thalassemia Ward of Lordegan Shohada Hospital. The study included transfusion-dependent patients with *β*-thalassemia major (β-TM) who received regular blood transfusions at Shohada Hospital.

To be eligible for participation, patients had to meet the following criteria: they were required to be diagnosed with β-TM, have a minimum 2-year history of using standard iron-chelating medications, be aged between 10 and 60 years, maintain a relatively stable average dose of iron chelators for at least the previous 3 months, and have serum ferritin levels of ≥1,000 μg/mL. Exclusion criteria included any change in their therapeutic regimen, such as switching the type of iron chelator, during the three-month study period. Additionally, patients were excluded if they were under 10 years of age, had hepatitis B or C, a history of a positive HIV test, chronic renal or cardiac failure, were undergoing iron chelation therapy with agents other than desferrioxamine, or were pregnant. Prior to group assignment, written informed consent was obtained from all eligible participants.

The study protocol was approved by the Medical Ethics Committee, assigned the ethics code IR.skums.rec.1394.278. All participants provided informed consent by signing a consent form prior to their enrollment in the study.

### 2.2 Study design and intervention

The randomized, double-blind trial was conducted between September 2023 and July 2024. Participants who met the inclusion criteria were assigned to either the interventional group or the control group. The interventional group received oak extract for a 12-week treatment period, while the control group was given a placebo capsule for the same duration. Participants in the intervention group were administered 300 mg of oak extract per day in a single daily dose.

The selected dose of 300 mg/day was based on prior human studies demonstrating safety and efficacy. Given that polyphenol absorption and metabolism are not significantly affected by age in the studied range (10–60 years), a uniform dose was considered appropriate.

All tablets, including both the active drug and placebo, were supplied by Goldaru and Soha Jissa Pharmaceutical Companies (located in Isfahan and Mazandaran, Iran, respectively). The tablets containing the active drug were formulated with 300 mg of oak extract. The placebo tablets were identical in size, color, shape, and smell to the oak extract tablets but did not contain the active ingredient.

Participants were advised to maintain their usual dietary habits throughout the study, and their intake of high-fat and high-sugar foods was assessed at baseline and post-intervention.

### 2.3 Sample size

Based on previous studies (21, 22), the total number of subjects needed for a two-way parallel trial to detect a treatment effect (*δ*) of 0.25, with a standard deviation of 0.55, at a 5% significance level and 80% statistical power, was calculated to be 77 patients. For a 2 × 2 crossover trial to detect the same effect (δ), the required sample size was determined using the formula: Crossover sample size = (1–r) × parallel sample size / 2, where *r* represents the correlation coefficient between repeated measurements of the primary endpoint in a crossover trial. Assuming a correlation coefficient (*r*) of 0.2 and accounting for a 10% dropout rate, the total number of participants needed for our crossover study was estimated to be 35.

According to earlier studies (21, 22), a total of 60 participants was required for a two-way parallel trial to detect the specified treatment effect.

The sample size was determined using statistical power analysis based on previous studies. With an 80% power and a significance level of 0.05, the minimum required number of participants was calculated. Recruitment challenges and budget constraints were factors that limited further expansion of the sample size.

### 2.4 Randomization and blinding

The study drug was allocated to patients in consecutive order using numbered treatment packs containing either the active treatment or placebo tablets. The investigators, patients, and laboratory staff were all blinded to the treatment assignments, and the treatment code was only revealed after all patients had completed the study drug regimen and all laboratory assays were finalized. Blinding was further ensured by using a placebo that was identical in appearance, dosing frequency, and protocol to the oak extract tablets. To assess potential adverse events and side effects, structured interviews were conducted.

### 2.5 Baseline and clinical data collection

Baseline data collected included demographic information (age and gender), history of splenectomy (including the time elapsed since the procedure, if applicable), transfusion history, and details of current treatment regimens, such as iron-chelating therapies (with desferrioxamine, deferasirox, and/or deferiprone), hydroxyurea therapy, and a history of hepatitis C or diabetes mellitus.

To minimize confounding variables, patients taking multivitamins or lipid-modifying medications were excluded from the study.

### 2.6 Specimen collection and analysis

In this study, fasting venous blood samples were collected from each patient on two occasions: once before the administration of a new blood transfusion and the initiation of medication, and again after 3 months at the conclusion of the study. The clot samples were allowed to sit at room temperature for 30 min, then centrifuged at 2500 rpm for 10 min to separate the serum, which was subsequently stored at −20°C until further analysis (29, 30). The lipid profile, including triglycerides, cholesterol, and HDL-C (all measured in mg/dL), as well as liver enzymes (ALT and AST, measured in units/L), were analyzed using a BT3000 autoanalyzer and commercial kits from Pars Azmoun. The levels of ferritin and hemoglobin in the patients’ blood were assessed using an ELISA kit (Monobind, Germany) and a Sysmex device (KX-21), with ferritin expressed in ng/ml.

### 2.7 Statistical analysis

The results were analyzed using the SPSS statistical software (version 24, Chicago). Data were presented as mean ± standard deviation (SD). The normality of the data distribution was assessed using the Kolmogorov–Smirnov test. For comparing normally distributed data between the two groups, an independent *T*-test was applied. Comparisons of data within each group, from the beginning to the end of the study, were performed using a paired *T*-test. For data that did not follow a normal distribution, the Kruskal-Wallis non-parametric test and the Mann–Whitney test were used. A *p*-value of less than 0.05 was considered statistically significant.

### 2.8 Encapsulation of oak extracts

The spray drying technique utilizes specialized equipment to create particles from a dispersion of active compounds within a solution containing a coating agent. Initially, a liquid formulation comprising a coating agent and an active ingredient dissolved in a solvent is atomized into droplets. This is achieved either through a nozzle using compressed gas or a rotary atomizer that employs a high-speed rotating wheel. Subsequently, a heated process gas, such as air or nitrogen, is introduced via a gas disperser to contact the atomized droplets, causing the solvent to evaporate. As the liquid rapidly evaporates from the droplets, particles are formed and settle at the bottom of the chamber. The resulting oak extract powder is then collected from the exhaust gases using a cyclone or bag filter. This method was implemented in a manner similar to the study conducted by Vehring (31).

### 2.9 Safety and efficacy assessments

Safety was evaluated by monitoring adverse events and assessing laboratory parameters, including complete blood count, hemoglobin, hematocrit, and liver function tests. Serum ferritin levels were measured at baseline and subsequently every 3 months. Ferritin levels were determined using commercial ELISA kits, kindly provided by Monobind. The C-reactive protein (CRP) status for each patient was also assessed as either positive or negative.

Potential adverse effects were monitored through structured patient interviews and laboratory evaluations, including liver function tests (ALT, AST) and complete blood count assessments.

### 2.10 Safety of Quercus therapy

Among patients receiving Quercus, the treatment was generally well tolerated, with a high compliance rate exceeding 90%. Compliance was determined through patient self-reporting and by counting the number of unused capsules returned to the clinic at each visit. No serious adverse drug reactions were observed, and there were no instances of patient withdrawal or treatment discontinuation due to drug-related adverse events.

## 3 Results

**Figure 1:**
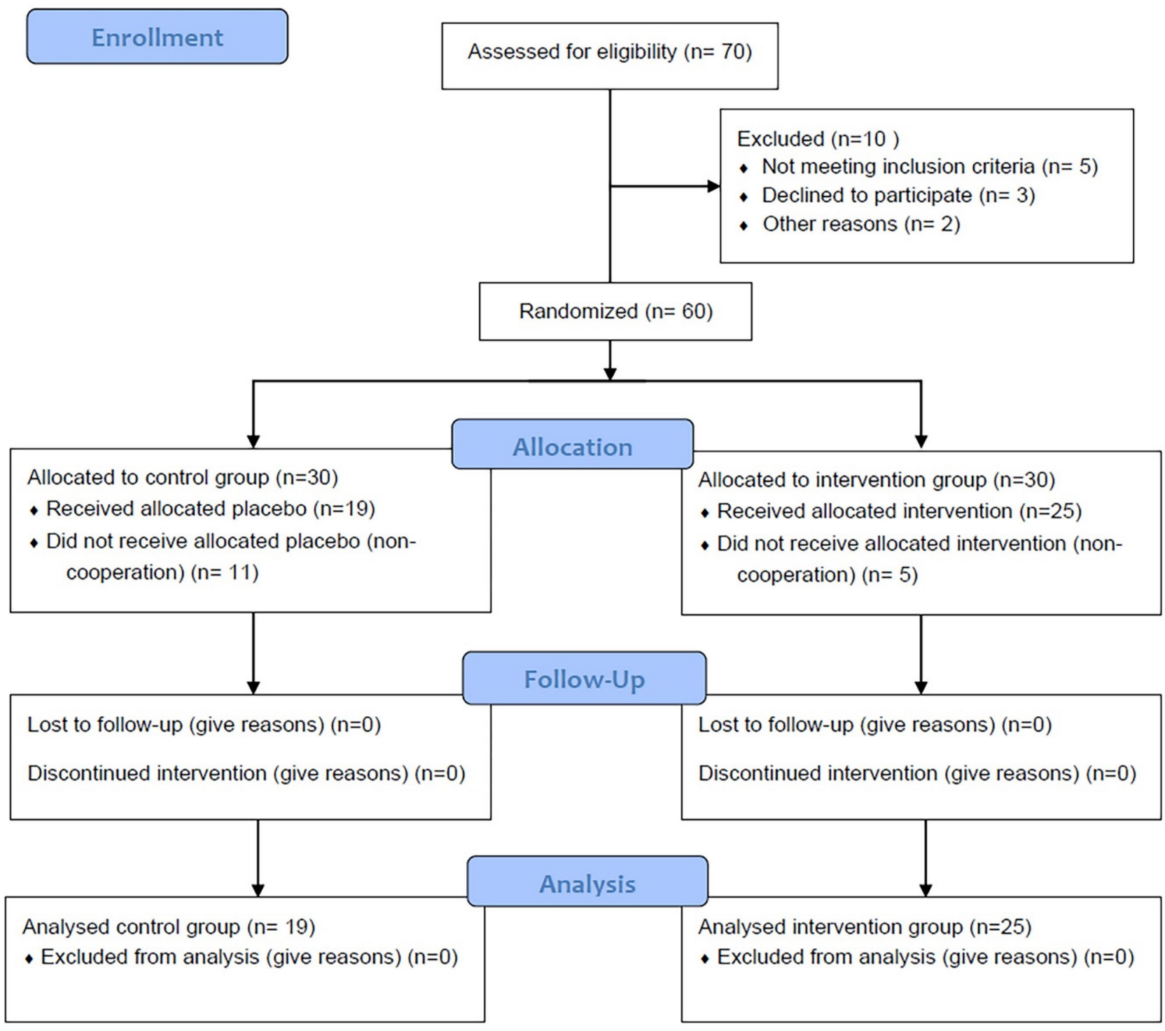
The flow diagram of the sampling process.

Between September 2023 and July 2024, a total of 105 patients with beta-thalassemia major were monitored at the Division of Thalassemia, Shohada Hospital. Out of these patients, 45 did not meet the inclusion criteria for the study. Of the remaining 60 patients who were enrolled in the two-period crossover trial, five from the Quercus group and eleven from the placebo group were lost to follow-up during the first period. Ultimately, 44 patients successfully completed the 3-month study (intervention group: *n* = 19; placebo group: *n* = 25; see Figure 1 and Table 1).

**Table 1:** Baseline demographic characteristics of patients, clinical characteristics of b-thalassemia major patients (average age, sex, ferritin, and hemoglobin of patients in each group).

Variables	Baseline		*p*-value
	Quercus (*n* = 19)	Placebo (*n* = 25)	
Age (year)	18.8 ± 4.9	20.4 ± 6.8	0.4^a^
Gender (M/F ratio)	51.4%	45.7%	0.57^b^
Hb (g/dL)	7.8 ± 2.1	7.5 ± 2.7	0.43^a^
Ferritin (ng/dL)	2,727 ± 2,261	2,725 ± 2,450	0.895^a^

a *p*-values less than 0.05 are considered statistically significant (comparison of mean and standard deviation between groups using the Independent *t*-test).

b *p*-values less than 0.05 are considered statistically significant (comparison between the intervention and control groups before and after the intervention using the Chi-Square test).

In the control group, the average triglyceride (TG) level after 3 months (184 mg/dL) showed a significant increase compared to the baseline level (167 mg/dL) and the average TG level in the group treated with oak extract (161 mg/dL) (*p* < 0.05). According to the findings, the mean cholesterol level in the intervention group (treated with oak extract) decreased from 125 mg/dL at baseline to 112 mg/dL after 3 months (*p* < 0.03). Conversely, the initial levels of cholesterol (124 mg/dL) and TG (167 mg/dL) in the control group did not differ significantly from the initial levels in the oak extract-treated group (TG = 161 mg/dL, *p* = 0.91; cholesterol = 125 mg/dL, *p* = 0.86), as shown in Table 2.

**Table 2:** Lipid profile in both control and case groups before and after the study.

Index	(Before/After)	Quercus (*n* = 19)	Placebo (*n* = 25)	*p*- value^a^
Triglycerides (mg/dl, mean ± SD)	Before	161 ± 76	167 ± 90	0.86
	After	160 ± 66	184 ± 85	0.74
*p*-value^b^		0.954	0.032	
Cholesterol (mg/dl, mean ± SD)	Before	125 ± 35	124 ± 20	0.30
	After	112 ± 30	124 ± 23	0.61
*p*-value^b^		0.003	0.930	
HDL-C (mg/dl, mean ± SD)	Before	26 ± 5	24.8 ± 12	0.30
	After	22 ± 7	20.4 ± 11	0.61
*p*-value^b^		0.008	0.016	
LDL-C (mg/dl, mean ± SD)	Before	63 ± 30	65 ± 33	0.24
	After	58 ± 27	56 ± 30	0.01
*p*-value^b^		0.17	0.79	

a *p*-values less than 0.05 are considered statistically significant (comparison of means and standard deviations between groups using the Independent *t*-test).

b *p*-values less than 0.05 are considered statistically significant (comparison of means and standard deviations within groups using the Paired *t*-test).

**Figure 2:**
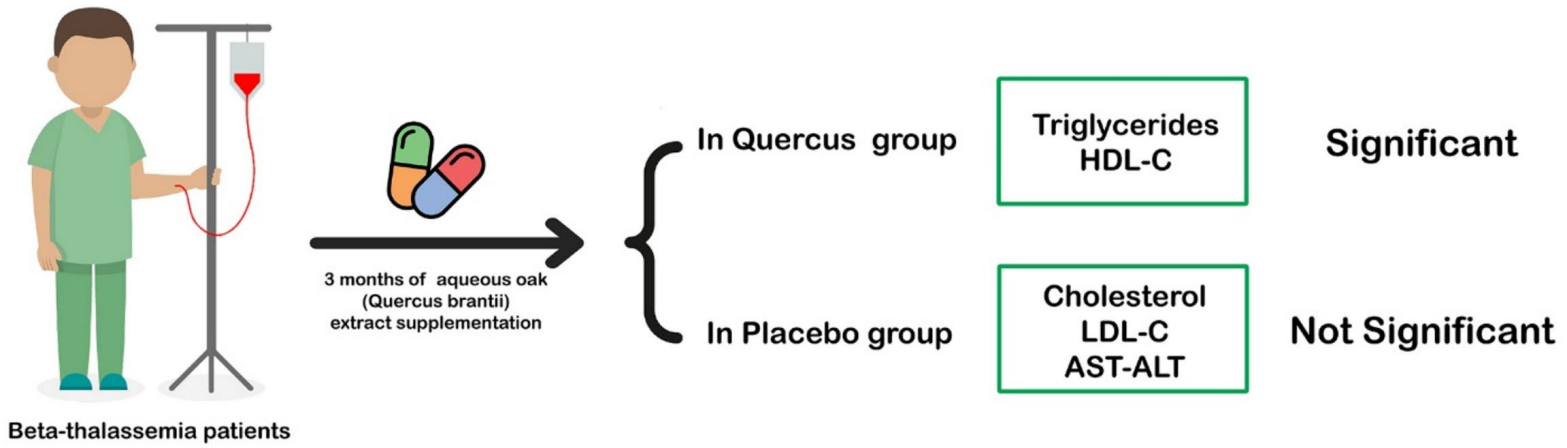
With aqueous Quercus extract cholesterol, HDL, and AST levels in the intervention group showed a significant decrease in patients with β-thalassemia major.

HDL levels decreased in both groups, which aligns with prior findings in beta-thalassemia patients. However, the reduction was not significantly different between the intervention and control groups (see Figure 2 and Table 3).

**Table 3:** Liver enzymes in both control and case groups before and after the study.

Index	(Before/After)	Quercus (*n* = 19)	Placebo (*n* = 25)	*p*-value^a^
AST (Unit/liter, mean ± SD)	Before	40.1 ± 23.1	44 ± 23	0.69
	After	38 ± 30	43 ± 29	0.75
*p*-value^b^		0.05	0.88	
ALT (Unit/liter, mean ± SD)	Before	24 ± 17	23 ± 11	0.17
	After	27 ± 14	24 ± 13	0.76
*p*-value^b^		0.42	0.81	

a *p*-values less than 0.05 are considered statistically significant (comparison of means and standard deviations between groups using the Independent *t*-test).

b *p*-values less than 0.05 are considered statistically significant (comparison of means and standard deviations within groups using the Paired *t*-test).

## 4 Discussion

Although many patients have been cured through bone marrow transplantation, a significant number still require regular blood transfusions (32). These transfusions help extend the lifespan of individuals with this condition, but lead to complications due to iron overload. Elevated iron levels, linked to oxidative stress, play a key role in liver apoptosis, tissue damage, and alterations in fatty acid synthesis and lipid profiles, all of which are attributed to insufficient antioxidant defenses (3).

### 4.1 Effects of Iranian oak extract (*Quercus brantii*) on liver enzymes

The liver, with its enzymatic and non-enzymatic antioxidants, serves as one of the body’s primary defense organs against oxidative compounds. However, excessive iron deposition in the liver, in the form of ferritin and hemosiderin, damages liver tissue and progressively elevates liver function markers such as alanine transaminase (ALT) and aspartate transaminase (AST) (33). These markers were observed in both groups during the study, with no significant difference in liver function, indicating no liver tissue damage over the 3-month period. Furthermore, the consumption of 300 mg/day of oak extract did not result in liver toxicity. Horvathova et al.’s (34) clinical trial also concluded that 300 mg of oak extract (administered in three 100 mg doses) did not cause liver complications, and our findings support the absence of side effects from this dose of oak extract.

**Figure 3:**
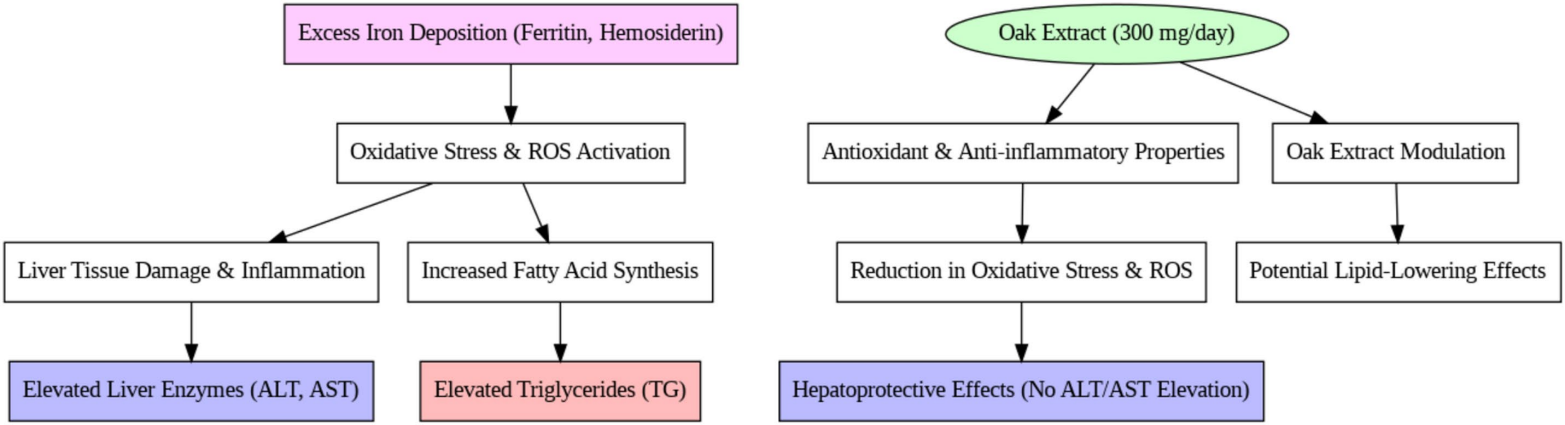
Mechanism of Iranian oak extract on liver enzymes metabolism.

Lipid abnormalities in beta-thalassemia have been noted in various reports, although the mechanisms behind these disorders remain unclear. In our study, the control group showed a significant increase in serum triglyceride (TG) levels over 3 months, which is consistent with previous studies on thalassemia patients. In 2004, Furukawa et al. (35) reported that elevated ROS (Reactive Oxygen Species) activity and NADPH oxidase systems in thalassemia patients lead to increased fatty acid production and expression of lipogenic genes, such as fatty acid synthetase (35). Similarly, Suman et al. (36) attributed the rise in triglycerides to liver damage and hormonal defects in thalassemia patients. These findings are also supported by studies conducted by Shams et al. (3) and Boudrahem-Addour et al. (37), who suggested that the increase in TG in beta-thalassemia major is due to oxidative stress and impaired antioxidant activity (see Figure 3).

### 4.2 Effects of Iranian oak extract (*Quercus brantii*) on lipid profile

In contrast, the intervention group treated with oak extract did not experience an increase in TG levels after 3 months of treatment. Pancreatic lipase plays a vital role in the digestion and absorption of TG from the small intestine, and certain plants, rich in tannins and phenolic compounds, inhibit enzymes like lipase. Oak extract contains 50–70% polyphenolic compounds (tannins, gallic acid, and ellagic acid) that bind to proteins and interfere with their function (38). These compounds possess antioxidant properties that significantly reduce oxidative stress and inhibit the production of intermediates in the fatty acid synthesis pathway. The absence of a TG increase in the intervention group suggests the positive effect of oak extract on this serum marker. These results align with the findings of Gholamhoseinian et al. (39), who studied the impact of oak extract on lipid profiles in animal models. Furthermore, studies involving tannin- and phenolic-rich plants have demonstrated reductions in TG levels due to enhanced antioxidant systems (40, 41). It is likely that the lack of TG increase in this study is due to the antioxidant properties of oak extract, which may inhibit pancreatic lipase and transporter enzymes, reducing TG absorption from the intestine.

Regarding cholesterol levels, no significant change was observed in the control group over the study period, while the intervention group exhibited a significant decrease in cholesterol levels (39). Plant sterols, widely recognized for their cholesterol-lowering effects, are often suggested as dietary interventions to reduce cholesterol absorption and plasma levels (42). Phytosterols, another class of compounds, disrupt cholesterol absorption and metabolism without causing adverse effects (43). Therefore, plants such as oak, which contain sterols and phytolipids, likely contribute to decreased cholesterol absorption and plasma levels (44). Since pancreatic cholesterol esterase and ACAT enzymes facilitate cholesterol absorption from intestinal villi (45), it is plausible that the inhibition of these enzymes by oak extract could be a contributing factor to the cholesterol reduction observed in this study. The reduction in total cholesterol in the intervention group is consistent with studies that used oak extract and other phenolic-rich plants to treat hyperlipidemia and fatty deposits in animals (39). Onakpoya et al.’s (46) meta-analysis further supported that tannins and phenolic compounds in green tea reduce cholesterol levels.

Nevertheless, several researchers have reported that cholesterol levels in thalassemia patients tend to be lower than in healthy individuals due to increased erythropoiesis and bone marrow activity (12, 37). In this study, cholesterol levels in the control group did not change significantly, but the reduction in the intervention group suggests that long-term oak extract consumption may amplify cholesterol reduction in these patients.

Another crucial factor in the lipid profile is HDL-C levels. In this study, HDL-C levels were evaluated in both control and intervention groups, and a significant decrease in HDL-C was observed in both. This decline is a well-known phenomenon in thalassemia patients and is caused by oxidative changes (47). Several studies, including those by Suman et al. (36), have similarly reported reductions in HDL-C levels among thalassemia patients due to increased paraoxonase 1 enzyme activity and iron-induced oxidation (48, 49). Shalev et al. (50) also noted that decreased HDL-C levels reflect increased internal cholesterol consumption by tissues and elevated oxidative stress (51, 52). Previous research has identified reduced HDL-C as a risk factor for cardiovascular complications in these patients (49, 52).

The reduction in HDL-C due to oxidative changes in thalassemia patients contributes to tissue damage (47). In individuals with iron overload, increased malondialdehyde levels in the liver indicate lipid peroxidation. Over the years, researchers have recognized the harmful effects of elevated lipid levels, such as increased cholesterol and decreased HDL-C, in atherosclerosis (12). In 2002, Omran (53) proposed that iron and TG increases contribute to LDL and HDL oxidation, leading to damage. Similar findings were observed in the article published by Pingali et al. (52–54).

In our study, while the reduction in HDL-C was slightly more pronounced in the intervention group than in the control group, this difference was not statistically significant. This finding, alongside the observed reduction in cholesterol, highlights the potential of oak extract in reducing total cholesterol. Since elevated cholesterol is a risk factor for atherosclerosis and cardiovascular diseases, oak extract may play a role in reducing the incidence of cardiovascular complications.

The observed HDL reduction aligns with previous reports in beta-thalassemia patients, which suggest that oxidative stress contributes to lower HDL levels. Further studies are required to determine the long-term impact of oak extract on HDL metabolism.

In this study, the increase in TG and ferritin levels in the control group was directly related, supporting the theory that elevated iron and TG levels contribute to the oxidation of LDL-C and HDL-C. Due to its antioxidant properties, enzyme inhibition, and potentially unknown pathways, oak extract appears to play a beneficial role in improving the lipid profile of these patients.

**Figure 4:**
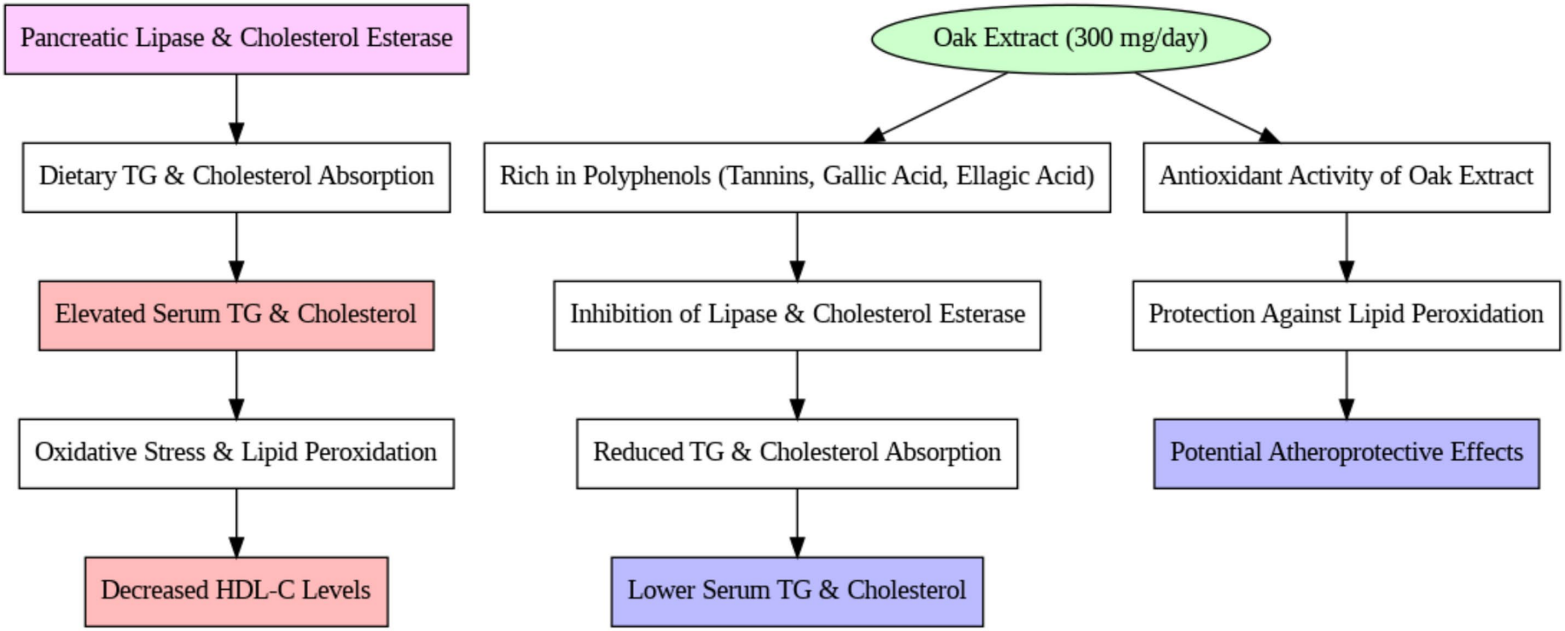
Mechanism of Iranian oak extract on lipid profile.

The observed cholesterol-lowering effect may be attributed to the polyphenolic content of oak extract, which inhibits pancreatic cholesterol esterase and ACAT enzymes involved in cholesterol absorption and metabolism (see Figure 4).

## 5 Conclusion

Positively affects lipid metabolism in beta-thalassemia major patients. The aqueous extract of Iranian oak demonstrated a significant reduction in total cholesterol levels but did not alter overall lipid metabolism. Further studies are needed to assess its long-term effects on HDL cholesterol. Specifically, the oak extract significantly reduced total cholesterol levels and prevented the increase in triglycerides, which are common complications in patients with iron overload. These results suggest that the antioxidant properties of the oak extract, likely due to its high content of flavonoids and polyphenolic compounds, can help mitigate oxidative stress and regulate lipid metabolism in these patients.

Although the study did not observe significant changes in liver enzymes (ALT, AST), the lack of liver toxicity further supports the safety of using oak extract as a potential adjunct therapy for managing lipid abnormalities in beta-thalassemia. The significant decrease in cholesterol levels and the stabilization of triglycerides highlight the potential cardiovascular benefits of this intervention.

However, given the limited sample size and the relatively short duration of the study, further research is necessary to confirm these findings and explore the long-term effects of oak extract supplementation. Future studies with larger and more diverse populations, as well as extended follow-up periods, are recommended to establish the broader clinical applications of oak extract in the management of lipid profiles and overall health in beta-thalassemia patients.

### 5.1 Strengths and limitations

### 5.2 Strengths

1. *Innovative Approach*: The study investigates the use of oak extract, which contains flavonoid and polyphenolic compounds, in treating lipid profile abnormalities in beta-thalassemia patients. This novel approach provides a potentially natural and cost-effective intervention for managing lipid metabolism and cardiovascular risks in these patients.
2. *Clinical Relevance*: The research addresses a significant clinical challenge—managing iron overload-induced lipid abnormalities in beta-thalassemia major patients. The findings could offer new therapeutic options that improve patients’ lipid profiles and reduce cardiovascular complications.
3. *Randomized Controlled Design*: The study’s randomized, double-blind, placebo-controlled design enhances the reliability of the results and minimizes biases, ensuring that any observed effects can be attributed to the intervention rather than external factors.
4. *Comprehensive Data Collection*: The study monitors several important biomarkers, including triglycerides, cholesterol, HDL-C, and liver enzymes, providing a well-rounded view of the intervention’s impact on both lipid metabolism and liver function.
5. *Safety Considerations*: The study emphasizes the safety of the intervention, confirming no significant adverse effects or liver toxicity from the 300 mg/day dosage of oak extract. This is crucial in clinical trials to ensure patient safety.

### 5.3 Limitations

1. *Limited Sample Size*: The relatively small sample size (44 patients completing the study) may limit the generalizability of the findings. A larger sample size would increase the study’s statistical power and allow for broader applicability of the results.
2. *Short Study Duration*: The study period of 3 months may be insufficient to fully assess the long-term effects of oak extract on lipid metabolism and cardiovascular risk. Future studies with extended follow-up periods would help determine the sustained impact of the intervention.
3. *Lack of Diversity in Patient Population*: The study was conducted in a specific population of beta-thalassemia patients in Iran. It would be beneficial to replicate the study in diverse populations to ensure the generalizability of the results across different demographic and genetic backgrounds.

## Data Availability

The original contributions presented in the study are included in the article/supplementary material, further inquiries can be directed to the corresponding author.
